# An unusual complication of a ventriculoperitoneal shunt: Endoscopic ultrasound-guided drainage of a giant cerebrospinal fluid pseudocyst

**DOI:** 10.1055/a-2218-2516

**Published:** 2024-01-09

**Authors:** Jayanta Samanta, Jahnvi Dhar, Pardhu Bharath Neelam, Nitish Sachdeva, Rishav Aggarwal, Antriksh Kumar, Antonio Facciorusso

**Affiliations:** 1 29751 Gastroenterology, Post Graduate Institute of Medical Education and Research, Chandigarh, India; 2 18567 Department of Medical and Surgical Sciences, Foggia University Hospital, Foggia, Italy

A 24-year-old man presented with a gradually progressive abdominal distension over 6 months. He received bilateral ventriculoperitoneal shunts 4 years back for tubercular meningitis with multiple revisions since then. At presentation, he had a functioning right ventriculo-pleural shunt and a blocked left ventriculoperitoneal shunt with right double J stent. Examination revealed markings of bilateral shunts in the neck with gross abdominal distension ( Fig. 1 ). A contrast-enhanced computed tomography scan of the abdomen revealed a large, encysted collection (16 × 25.6 × 41 cm) displacing the bowel loops peripherally, no free fluid, and a left ventriculoperitoneal shunt entering the collection ( Fig. 2 ) **,** also visualized on endoscopic ultrasound (EUS) ( Fig. 3 ) **.** The neurosurgery team planned to remove the blocked ventriculoperitoneal shunt only after drainage of the collection to prevent further fluid accumulation. Exploring different treatment options in a multidisciplinary team meeting, the patient opted for EUS-guided drainage.

**Fig. 1:**
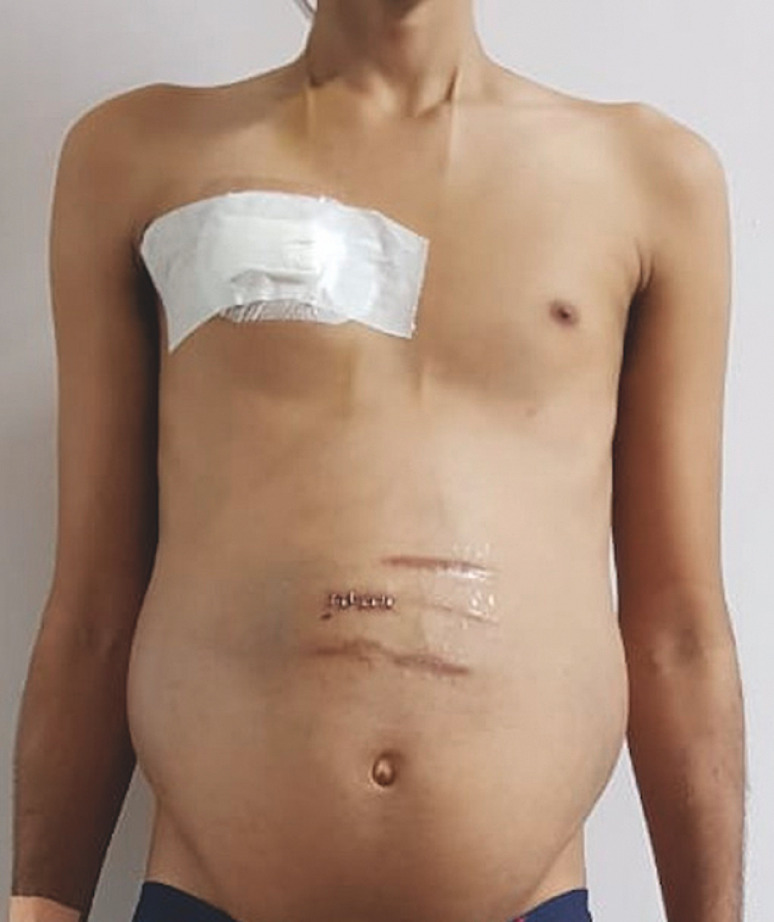
Pre-procedure photograph with markings of bilateral shunts in the neck and scar marks of previous surgeries, with gross abdominal distension. The white bandaged area on the right chest is of the recent ventriculo-pleural shunt procedure.

**Fig. 2:**
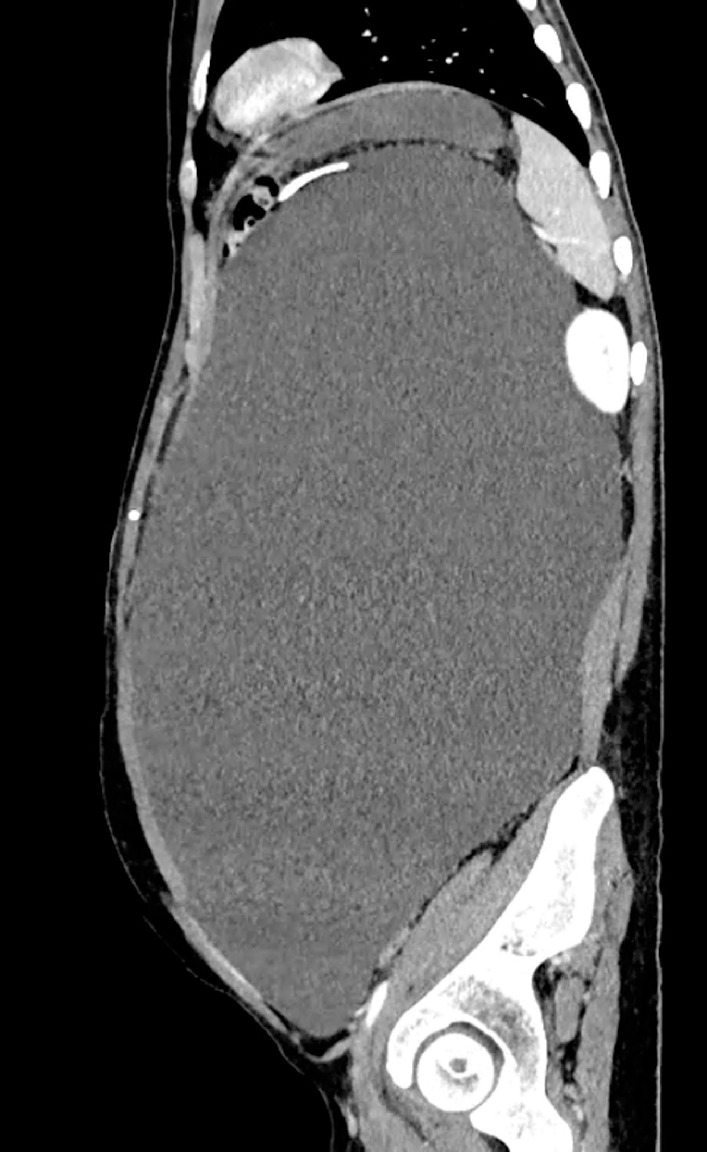
Computed tomography (CT) scan showing a large, encysted collection (16 × 25.6 × 41 cm) occupying the whole abdomen, displacing the bowel loops peripherally, with no free fluid and a left ventriculoperitoneal shunt entering the collection.

**Fig. 3:**
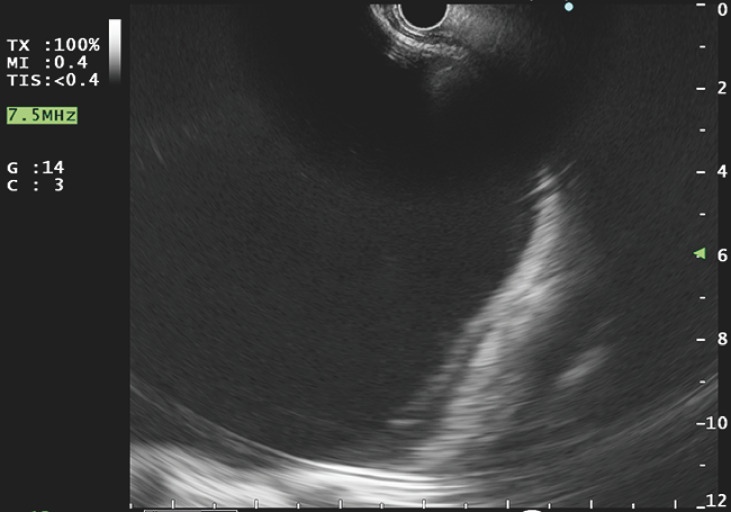
Endoscopic ultrasound image of a large anechoic cystic collection with a left ventriculoperitoneal shunt entering it.

Under fluoroscopic and endoscopic guidance, standard steps of an EUS-guided cysto-enterostomy were followed (assessment using linear echoendoscope [GIF UCT180; Olympus, Tokyo, Japan], puncture with a 19-G needle [EZ Shot3 Plus; Olympus Medical], fluid aspiration, guidewire passed and coiled within the cavity, tract dilatation using an 8.5-Fr cystotome [CYSTO085U; G-Flex, Nivelles, Belgium]), and a 10-Fr nasocystic drain was passed deep down in the pelvis ( Video 1 ) **.** Post-procedure, 11 liters of fluid was drained (cerebrospinal fluid [CSF]: lymphocytic, high protein, gene-expert negative, sterile, positive beta-2 transferrin) and distension decreased remarkably. A post-procedure X-ray of the abdomen was done ( Fig. 4 ) along with CT, which revealed complete resolution ( Fig. 5 ); the left ventriculoperitoneal shunt was removed subsequently. Thereafter, the nasocystic drain was removed under endoscopic guidance and the patient discharged, with no recurrence 1 year post-procedure.

**Fig. 4:**
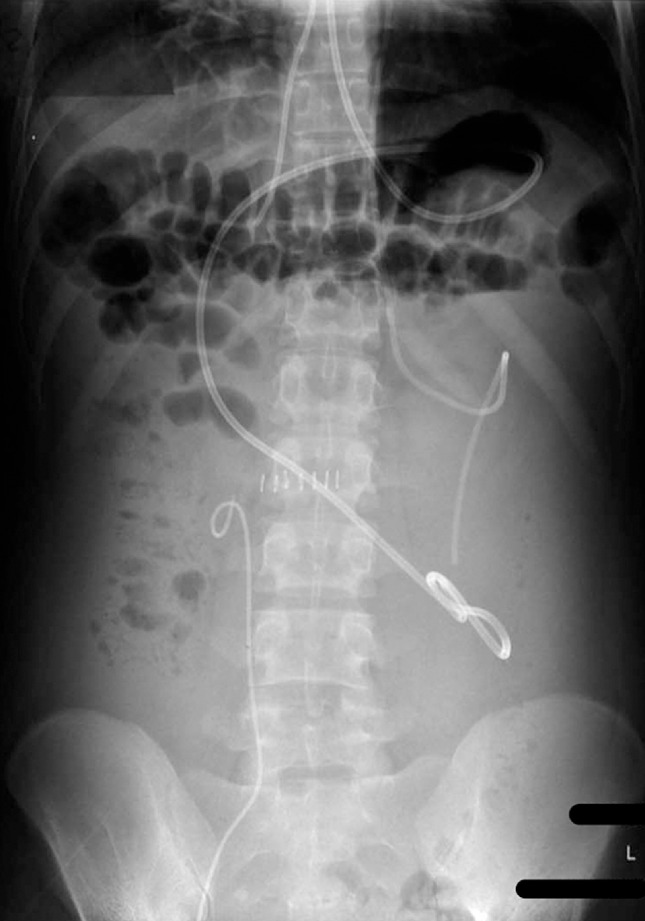
Post-procedure X-ray of the abdomen showing a nasocystic drain, right ventriculo-pleural shunt, left ventriculoperitoneal shunt, and right double J stent.

**Fig. 5:**
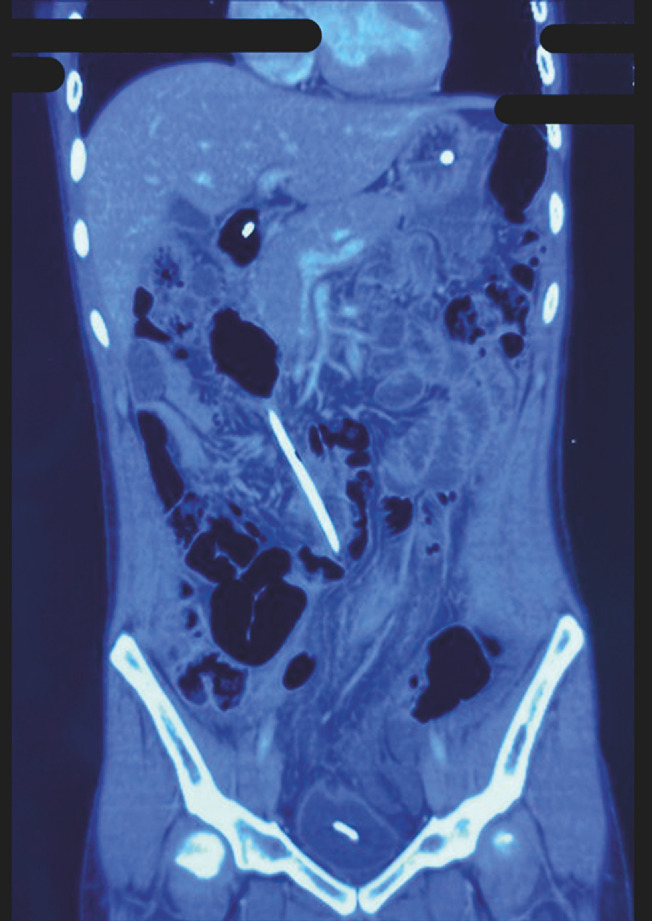
Post-procedure CT of the abdomen showing complete resolution of the cerebrospinal fluid pseudocyst with the nasocystic drain lying in the pelvis.

Endoscopic ultrasound-guided drainage of a giant (41 cm) cerebrospinal fluid pseudocyst using a nasocystic drain, leading to complete resolution of a cystic collection.Video 1

A ventriculoperitoneal shunt is fraught with intraabdominal complications (10–25%), among which abdominal CSF pseudocysts are extremely rare (0.3–6.8%), life-threatening, and occurring 3 weeks to 21 years post-procedure. Consensus is lacking regarding their management 1 2 3 4 . To address the issue of transmural contamination with EUS-drainage, we used two strategies: 1) dilatation of the tract with an 8.5-Fr cystotome with placement of a 10-Fr nasocystic drain to avoid over-dilatation; and 2) choosing a nasocystic drain over a transmural pigtail stent to drain the fluid outside, negating the chances of retro-contamination from intestinal contents 5 .

To the best of our knowledge, this is the first report of EUS-guided drainage of such a giant CSF pseudocyst and appears to be a safe and effective alternative.

Endoscopy_UCTN_Code_TTT_1AS_2AC

## Notes

Endoscopy E-Videos https://eref.thieme.de/e-videos
*E-Videos* is an open access online section of the journal *Endoscopy* , reporting on interesting cases and new techniques in gastroenterological endoscopy. All papers include a high-quality video and are published with a Creative Commons CC-BY license. Endoscopy E-Videos qualify for HINARI discounts and waivers and eligibility is automatically checked during the submission process. We grant 100% waivers to articles whose corresponding authors are based in Group A countries and 50% waivers to those who are based in Group B countries as classified by Research4Life (see: https://www.research4life.org/access/eligibility/ ). This section has its own submission website at https://mc.manuscriptcentral.com/e-videos .
